# Phenotypic Identification and Fine-Mapping of the Rice Narrow-Leaf Mutant *nal25*

**DOI:** 10.3390/plants14162528

**Published:** 2025-08-14

**Authors:** Kaizhen Xie, Fuan Niu, Peng Hu, Can Cheng, Huangwei Chu, Jihua Zhou, Bin Sun, Yuting Dai, Liming Cao, Anpeng Zhang

**Affiliations:** 1 Key Laboratory of Germplasm Innovation and Genetic Improvement of Grain and Oil Crops (Co-Construction by Ministry and Province), Ministry of Agriculture and Rural Affairs, Crop Breeding and Cultivation Research Institute, Shanghai Academy of Agricultural Sciences, Shanghai 201403, China; 18621311381@163.com (K.X.); niufuan@saas.sh.cn (F.N.); chengcan@saas.sh.cn (C.C.); chuhuangwei@saas.sh.cn (H.C.); zhoujihua@saas.sh.cn (J.Z.); sxb0708@126.com (B.S.); daiyuting@saas.sh.cn (Y.D.); 2 Hubei Key Laboratory of Food Crop Germplasm and Genetic Improvement, Institute of Food Crops, Hubei Academy of Agricultural Sciences, Wuhan 430064, China; 15168331324@126.com

**Keywords:** rice, narrow-leaf mutant, fine-mapping, *NAL25*

## Abstract

Leaf morphology significantly impacts rice (*Oryza sativa* L.) plant architecture and yield. Here, we identified and characterized a novel narrow-leaf mutant, *nal25*, derived from indica rice cultivar ‘Huazhan’ using EMS mutagenesis. Phenotypic analyses revealed that *nal25* exhibited significantly narrower leaves, reduced plant height, increased tiller number, and notably decreased grain size, seed setting rate, and thousand-grain weight compared to the wild type. Genetic analyses demonstrated that the narrow-leaf phenotype is controlled by a single recessive nuclear gene. Through precise localization analysis, the *NAL25* gene was located within a region of approximately 103 kb on the long arm of rice chromosome 7. The sequencing results showed that the mutant *nal25* had a T to C mutation at position 173 of the heat-shock protein gene *LOC_Os07g09450* encoding the DnaJ domain in this interval, resulting in a change in amino acid 58 from leucine to proline. The qRT-PCR results showed that the expression level of *NAL25* gene decreased in the mutant. The *nal25* mutant obtained in this study exhibits stable mutant phenotypes, including dwarfism and excessive tillering, traits typically unfavorable for rice production. Nevertheless, it serves as valuable genetic material for forward genetics approaches to identify yield-related genes regulating leaf morphology and culm height. Thus, research on the *nal25* mutant advances the development of rice varieties with ideal plant architecture, thereby stabilizing yield increases and safeguarding global food security.

## 1. Introduction

Rice (*Oryza sativa* L.) is one of the world’s most important staple crops, serving as the primary food source for approximately 3.5 billion people globally. With the continuous rise in population, decreasing availability of arable land, and increasing environmental pressures, sustaining rice production presents significant challenges. Thus, maintaining stable rice yields is essential for ensuring global food security [1,2]. Currently, optimizing plant architecture is widely recognized as a critical strategy for breeding high-yielding rice varieties. Rice plant architecture is primarily determined by the morphological characteristics and spatial arrangements of the roots, stems, leaves, and panicles [3]. Among these, leaves play a pivotal role through photosynthesis, providing energy and organic materials necessary for plant growth and development. Leaf morphology directly influences photosynthetic efficiency and the leaf area index (LAI), consequently affecting photosynthetic product accumulation, and ultimately, rice yield [4]. Therefore, manipulating rice leaf shape has emerged as a critical approach for improving plant architecture and enhancing grain yield.

Rice leaf shape traits mainly include leaf length, width, color, thickness, rolling, drooping, and leaf angle [5]. An optimal balance between leaf length and width is particularly crucial, as it maximizes leaf area and enhances light utilization efficiency [6]. Previous studies have demonstrated that genes influencing rice leaf morphology mainly regulate leaf vein architecture, hormone synthesis and transport, and cellular proliferation or elongation [6]. Narrow-leaf mutants are valuable resources for dissecting the genetic mechanisms controlling leaf area in rice. Several narrow-leaf mutants, such as *nal1* [7], *nal2/3* [8], *nal7* [9], *nal9* [10], *nal11* [11,12], *nal21* [13], *nal22* [14], *dnl1* [15,16], *nsl2* [17], and *avb* [18], have been reported, all exhibiting narrower leaves and reduced plant height compared to wild types.

Genes associated with leaf width in rice have mainly been identified on chromosomes 3, 4, 6, 7, 11, and 12. For instance, several critical genes, including *NAL7*, *NAL9*, *NAL21*, *NAL22*, and *AVB*, are localized on chromosome 3. The *NAL7* gene encodes a flavin-containing monooxygenase, which is involved in leaf development and auxin biosynthesis [9]. *NAL9* encodes an ATP-dependent Clp protease proteolytic subunit involved in regulating cell division [10]. *NAL21* encodes ribosomal protein small subunit 3A (RPS3A), participating in auxin responses and thus regulating leaf development [13]. The *NAL22* gene encodes a Maf-like nucleoside triphosphate pyrophosphatase protein family member that is highly expressed in leaves and roots, regulating leaf width through the modulation of leaf vein diameter and cell division [14]. The *AVB* gene encodes a conserved protein of unknown function essential for maintaining normal cell division in rice [18].

On chromosome 4, *NAL1* encodes an unknown-function protein, predominantly expressed in vascular tissues, which is involved in auxin polar transport and leaf cell division [7]. *NSL2*, located on chromosome 6, encodes a small subunit of ribonucleotide reductase, affecting leaf development through nucleotide transport and metabolic regulation [17]. On chromosome 7, *NAL11* encodes a heat-shock protein (HSP) containing a DNAJ domain involved in cell cycle control, proliferation, chloroplast development, and gibberellin biosynthesis [12]. *NAL2* and *NAL3*, homologous genes located on chromosomes 11 and 12, respectively, encode WOX3A proteins belonging to the plant-specific Wushel-related homeobox (WOX) transcription factor family, regulating gene transcription and auxin transport during leaf development [8]. *DNL1*, located on chromosome 12, encodes a cellulose synthase-like D4 protein involved in gibberellin metabolism and leaf growth [16].

Additionally, several transcription factors regulate leaf growth by influencing cell proliferation or plant hormone biosynthesis. For example, the rice transcription factors *Growth-regulating factor 1* (*OsGRF1*) and *GRF-interacting factor 1* (*OsGIF1*) control leaf growth through cell-cycle-related gene expression [19]. *Auxin response factor 11* (*ARF11*) regulates leaf development by modulating brassinosteroid receptor gene expression [20].

Although narrow-leaf regulatory genes in rice frequently exhibit pleiotropic effects—influencing plant height, tillering, and stem thickness—the molecular mechanisms coordinating these interdependent morphological traits remain unresolved. This knowledge gap impedes the reconstruction of regulatory networks for leaf morphogenesis. To address this, we generated a stable narrow-leaf mutant *nal25* via EMS mutagenesis in indica rice ‘Huazhan’. Agronomic traits of *nal25* were systematically characterized, confirming its narrow-leaf phenotype. The mutant also displayed pleiotropic effects, including reduced plant height (dwarfism), increased tiller number, and decreased yield. Genetic analysis indicated that the narrow-leaf trait is governed by a single recessive gene. Subsequently, map-based cloning was employed to localize the target gene. The candidate gene was sequenced for validation, identifying the causal mutation site. Its expression pattern was preliminarily analyzed to investigate gene function. This study provides novel genetic resources for elucidating the molecular mechanisms underlying leaf shape development in rice. It advances research on ideal plant architecture (IPA) and establishes a foundation for achieving precision and efficiency in rice breeding.

## 2. Materials and Methods

### 2.1. Plant Materials

All experimental materials employed in this work originated from and were maintained in our laboratory stock collection. The indica rice cultivar ‘Huazhan’, the genetically stable EMS-induced narrow-leaf mutant *nal25* derived from ‘Huazhan’, and the japonica rice restorer line ‘Shenhui 26’ (SH 26) were used in this study. All plant materials were cultivated during the normal rice growing season at the Zhuanghang Experimental Base, Shanghai Academy of Agricultural Sciences (30°89′ N, 121°39′ E). Each genotype was planted with six rows, comprising six plants per row, at a standard spacing of 20 cm × 20 cm, ensuring a single-plant-per-hill planting. All plants were managed uniformly according to standard agricultural field practices.

### 2.2. Phenotypic and Agronomic Trait Analysis

At the heading stage, the length and width of flag leaves were measured in both the wild-type ‘Huazhan’ and the mutant *nal25*. At maturity, agronomic traits including plant height, number of effective panicles, panicle length, grain length, grain width, and thousand-grain weight were recorded. Ten plants exhibiting uniform growth vigor within each plot were randomly selected for measurement, and mean values were calculated. Statistical analyses were conducted using IBM SPSS statistics 22.0.

### 2.3. Genomic DNA Extraction and PCR

Genomic DNA was extracted from rice leaves using the CTAB method [21]. PCR amplifications were performed using the 2× Hieff^®^ PCR Master Mix (No Dye) (Yeasen Biotechnology Co., Ltd., Shanghai, China). The total reaction volume was 20 μL, consisting of 10 μL of 2× Hieff^®^ PCR Master Mix, 8.8 μL sterile ddH_2_O, 0.4 μL each of forward and reverse primers (10 μM), and 0.4 μL genomic DNA template. PCR conditions were as follows: initial denaturation at 94 °C for 5 min; followed by 35 cycles of denaturation at 94 °C for 30 s, annealing at 58 °C for 30 s, and extension at 72 °C for 45 s; with a final extension step at 72 °C for 10 min.

### 2.4. Genetic Analysis of the nal25 Mutant

The mutant *nal25* was crossed as the maternal parent with indica rice ‘Huazhan’ and japonica restorer line ‘SH26’ to generate two separate hybrid populations (*nal25*/Huazhan and *nal25*/SH26). The numbers of normal and mutant phenotypes were recorded in both F_1_ and F_2_ segregating populations. Data were statistically analyzed using IBM SPSS statistics 22.0, and chi-square tests were performed to evaluate the segregation ratio and inheritance pattern of the *nal25* mutation in the F_2_ populations.

### 2.5. Fine-Mapping of the NAL25 Gene

For initial genetic mapping, genomic DNA was extracted from approximately 2.0 g of fresh leaves from the parental lines (japonica ‘SH26’, mutant *nal25*, and their F_1_ progeny) and 21 mutant phenotype plants from the segregating F_2_ population. PCR amplification was performed using evenly distributed SSR markers covering the 12 rice chromosomes (primer sequences synthesized by Sangon Biotech Co., Ltd., Shanghai, China). PCR products were separated by 8% polyacrylamide gel electrophoresis (PAGE), visualized by silver staining (0.1% AgNO_3_ and 1.5% NaOH), and genotypes of each F_2_ mutant individual were analyzed. According to preliminary mapping results, sequence differences between indica and japonica genomes were identified by aligning genomic sequences using the NCBI database (accessed 31 March 2024; https://blast.ncbi.nlm.nih.gov/Blast.cgi). New primers for fine-mapping were designed using Primer Premier 5.0 software (Table 1). The F_2_ mapping population size was expanded for further fine-mapping. Open reading frames (ORFs) within the fine-mapping interval were retrieved from the rice genome database (accessed 20 June 2024; http://rice.uga.edu/cgi-bin/gbrowse/rice/), and candidate gene functions were predicted. Candidate genes within this region were then sequenced for validation.

### 2.6. Gene Expression Analysis of NAL25

Total RNA was extracted from rice leaves using the Polysaccharides & Polyphenolics-Rich Plant RNA Extraction Kit (Vazyme Biotech Co., Ltd., Nanjing, China). First-strand cDNA synthesis was performed using the HiScript III 1st Strand cDNA Synthesis Kit (+gDNA wiper) according to the manufacturer’s instructions. Real-time quantitative PCR (qPCR) assays were carried out using Taq Pro Universal SYBR qPCR Master Mix (Vazyme Biotech Co., Ltd.) on a QuantStudio 6 Flex Real-Time PCR System (Thermo Fisher Scientific, Waltham, MA, USA). Reaction conditions are detailed in Table 1. Rice actin was employed as the internal reference gene, and the primer sequences for qPCR analysis are provided in Table 2. All qPCR reactions were performed following the manufacturer’s recommended protocol.

## 3. Results

### 3.1. Agronomic Traits of the Narrow-Leaf Mutant nal25

**Figure 1:**
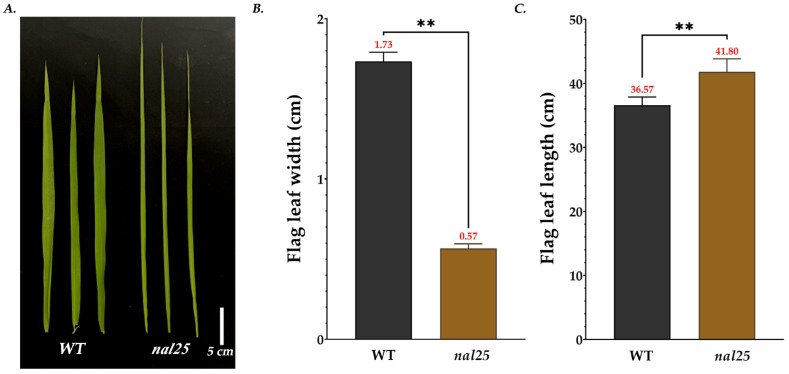
Flag leaf morphology of wild-type ‘Huazhan’ and mutant *nal25*. (**A**) Flag leaf morphology, scale bar = 5 cm; (**B**) comparison the length of flag leaf, ** significantly different at *p* < 0.01; (**C**) comparison the width of flag leaf, ** significantly different at *p* < 0.01.

**Figure 2:**
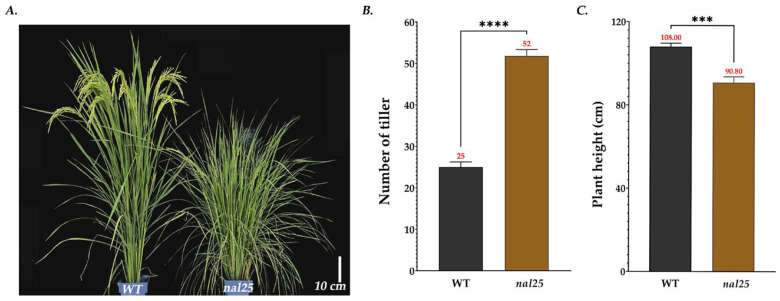
Plant phenotype of wild-type ‘Huazhan’ and mutant *nal25.* (**A**) Plant phenotype at heading stage, scale bar = 10 cm; (**B**) comparison of the number of tillers at heading stage, **** significantly different at *p* < 0.0001; (**C**) comparison of plant height at maturation stage, *** significantly different at *p* < 0.001.

The mutant used in this study was isolated from an EMS-mutagenized mutant library of the rice variety ‘Huazhan’ in previous work. Following multiple generations of self-pollination, its traits are stably inherited. Evidently, the leaf width was significantly reduced, measuring 0.57 ± 0.02 cm, corresponding to 33.14% of the wild-type leaf width (Figure 1C, Table 3). Conversely, the flag leaf of the main panicle of *nal25* presented a distinctive long and narrow phenotype (Figure 1A). Specifically, the flag leaf length of *nal25* was significantly greater, measuring 41.80 ± 1.66 cm, approximately 1.14 times that of the wild-type (Figure 1B, Table 3). Additionally, at the heading stage, compared with the wild-type cultivar ‘Huazhan’, the mutant *nal25* exhibited a significantly increased tiller number, averaging 25 ± 0.82 tillers, which was 2.08 times that of the wild-type (Figure 2A,B, Table 3). At maturity, the plant height of *nal25* was significantly reduced, averaging 90.80 ± 2.48 cm, about 84.07% of the wild-type height (Figure 2C, Table 3).

**Figure 3:**
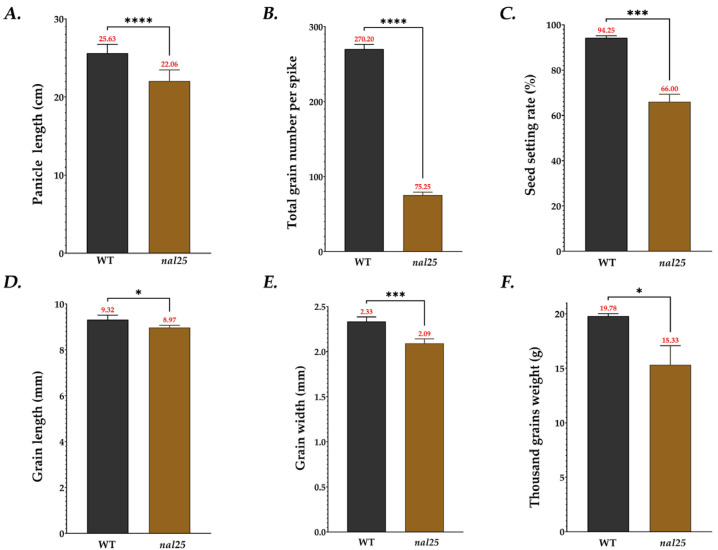
Comparison the agronomic traits of wild-type ‘Huazhan’ and mutant *nal25*: *(***A**) panicle length; (**B**) total grain number of per spike; (**C**) seed setting rate; (**D**) grain length; (**E**) grain width; (**F**) thousand-grain weight. * significantly different at *p* < 0.05; *** significantly different at *p* < 0.001; **** significantly different at *p* < 0.0001.

Other agronomic traits also significantly decreased compared with the wild-type, including panicle length, total grain number per panicle, seed setting rate, grain length, grain width, and thousand-grain weight (Figure 3, Table 3). Specifically, the panicle length was 22.06 ± 1.33 cm (86.07% of wild-type), total grain number per panicle was 75.25 ± 3.49 (27.84% of wild-type), seed setting rate was 66.00 ± 2.92% (70.03% of wild-type), grain length was 8.97 ± 0.09 cm (96.24% of wild-type), grain width was 2.09 ± 0.04 cm (89.69% of wild-type), and thousand-grain weight was 15.33 ± 1.52 g (77.50% of wild-type) (Figure 3, Table 3). Collectively, these results indicate that the *nal25* mutant exhibits not only significant morphological changes in leaf width, but also reduced yield potential.

### 3.2. Genetic Analysis of the Narrow-Leaf Mutant nal25

To determine the inheritance pattern of the *nal25* mutation, two cross-combinations were generated with the mutant *nal25* as the maternal parent crossed separately with indica cultivar ‘Huazhan’ and japonica restorer line ‘Shenhui 26’. The resulting F_1_ plants from both crosses exhibited a normal leaf phenotype. In the F_2_ generation obtained from selfing F_1_ individuals, segregation for leaf phenotype occurred, producing both normal and narrow-leaf plants.

In the cross *nal25*/Shenhui 26, among 1145 F_2_ plants, 862 exhibited normal leaves and 283 had narrow leaves, with a segregation ratio of 3.04:1.00 (χ^2^ = 0.05 < χ^2^_0.05_ = 3.84) (Table 4). Similarly, in the cross *nal25*/Huazhan, among 1192 F_2_ plants, 905 had normal leaves and 287 had narrow leaves, with a segregation ratio of 3.15:1.00 (χ^2^ = 0.46 < χ^2^_0.05_ = 3.84) (Table 4). Both segregation ratios fit a typical Mendelian inheritance pattern of 3:1, suggesting that the *nal25* mutation is recessive and controlled by a single nuclear gene.

### 3.3. Fine-Mapping and Candidate Gene Prediction of NAL25

For initial mapping of the *NAL25* gene, genomic DNA from parental lines, F_1_ plants, and 21 mutant individuals was analyzed using 48 SSR markers evenly distributed across the 12 rice chromosomes (Table S1). Based on polyacrylamide gel electrophoresis and band-pattern analyses, *NAL25* was mapped between markers RM7479 and R7M20 on chromosome 7.

**Figure 4:**
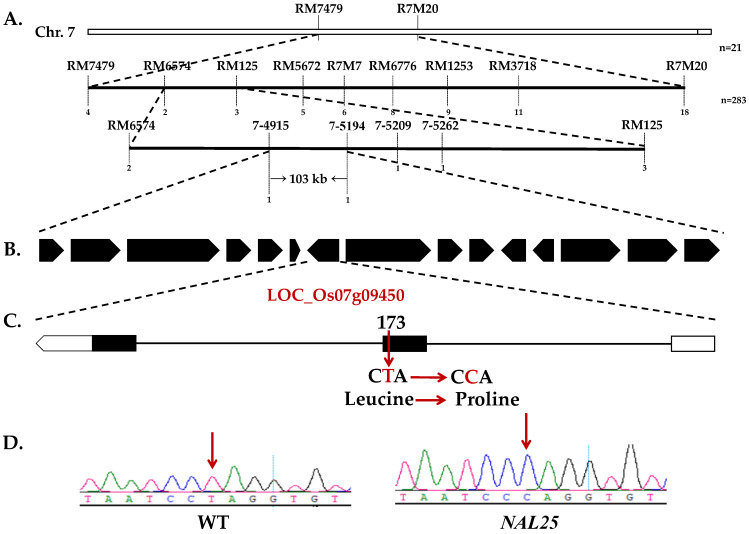
Fine-mapping of *NAL25.* Note: (**A**) fine-mapping of *NAL25* gene, *n* represents the number of single plants for mapping; (**B**) the open reading frame in the region; (**C**) the diagram of *NAL25* gene; (**D**) sequencing comparative analysis of wild-type.

To narrow this region, 283 F_2_ mutant plants were further analyzed using additional molecular markers located between RM7479 and R7M20. Fine-mapping localized *NAL25* between markers RM6574 and RM125. Subsequently, four insertion–deletion (InDel) markers 7-4915, 7-5194, 7-5209, and 7-5262 were developed within this interval, ultimately refining the target region to a 103 kb interval between markers 7-4915 and 7-5194 (Figure 4).

Within this 103 kb region, rice genome database searches identified 15 open reading frames (ORFs). Sequencing revealed a T→C single-nucleotide substitution in the candidate gene LOC_Os07g09450, causing an amino acid change from leucine to proline at position 170 (Figure 4). Importantly, no sequence variations (SNPs or indels) were identified within the other 14 genes in the interval upon comparison of mutant and wild-type sequences. This mutation represents a novel allele distinct from previously reported variants, likely affecting protein structure and function.

### 3.4. Expression Analysis of the NAL25 Gene

**Figure 5:**
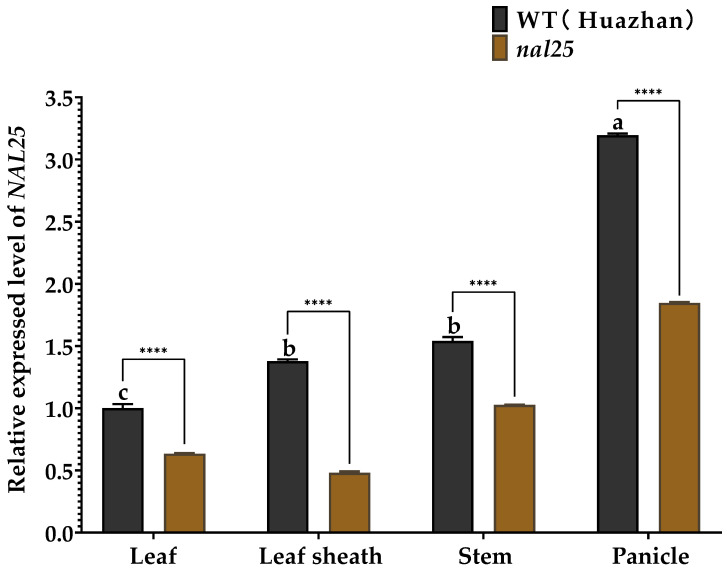
The expression level of the *NAL25* in the leaf, leaf sheath, stem, and spike. Note: **** significantly different at *p* < 0.0001.

Quantitative RT-PCR was conducted to examine the expression of LOC_Os07g09450 in leaves, leaf sheaths, stems at the tillering stage, and young panicles at 10 days after flowering in both wild-type ‘Huazhan’ and the mutant *nal25*. Expression analyses indicated that *NAL25* transcripts were detected in all tissues examined, with the highest expression in young panicles, followed by leaf sheaths and stems (Figure 5).

Notably, LOC_Os07g09450 expression was significantly reduced in all tissues of the *nal25* mutant compared to the wild-type. The mutant exhibited phenotypic traits such as shorter plant height, narrower leaves, and increased tiller number (Figure 1). qRT-PCR analysis revealed a significant decrease in the transcript abundance of this gene in mutant- compared to wild-type. This observation suggests that the mutation may affect post-transcriptional regulation of *LOC_Os07g09450*. Furthermore, significantly reduced thousand-grain weight and seed setting rate were observed in *nal25*, implying that LOC_Os07g09450 may regulate rice yield.

## 4. Discussion

In the late 1990s, Yuan Longping, widely recognized as the “father of hybrid rice”, highlighted that ideal super-high-yield rice should possess a distinctive leaf phenotype described as “long, erect, narrow, concave, and thick” in three major functional leaves (flag leaf, second leaf, and third leaf from the top). This specific leaf morphology maximizes photosynthetic efficiency, thus providing abundant resources for high-yield rice production [22]. Narrow-leaf rice mutants frequently exhibit additional phenotypic alterations, such as reduced plant height, increased tiller numbers, hull splitting, fewer effective panicles, and decreased thousand-grain weight [6,7,10]. Consequently, exploring the molecular mechanisms underlying rice narrow-leaf formation is critical for designing ideal plant architectures and breeding super-high-yield rice varieties.

This study identified a novel EMS-induced mutant, *nal25*, derived from the indica rice cultivar ‘Huazhan’. Compared to wild-type plants, the *nal25* mutant exhibits pleiotropic phenotypic alterations, including significantly reduced plant height, narrower and elongated leaves, and an increased number of effective panicles. However, these potentially advantageous traits are counterbalanced by substantial yield-compromising defects, namely reduced panicle length, fewer grains per panicle, lower seed set rate, diminished grain dimensions (length and width), and decreased 1000-grain weight, collectively leading to severely impaired final yield. This narrow-leaf trait is controlled by a single recessive gene, as shown by genetic analysis of hybrid combinations of mutant *nal25*/’Huazhan’ and mutant *nal25*/’Shenhui26’. Fine-mapping analysis revealed that *NAL25* was located in a 103 kb region of chromosome 7 in rice. Sequencing alignment revealed a single-base mutation at position 173 from the T to C mutations of the *LOC_Os07g09450* gene, resulting in an amino acid substitution from leucine to proline. At the same time, expression analysis revealed that the expression level of this gene was significantly downregulated in the leaves, sheaths, stems, and young spikes in mutant *nal25*. *NAL25* is a new allele of *NAL11*, encoding the OsHSP40 protein of rice, and participates in the regulation of rice leaf morphology development and the biosynthesis of gibberellin [12]. This gene has been reported to possess three allelic forms, namely *ZYX-nal11* [11], *lw7* [23], and *NAL11^−923del−1552^* [12]. In the *ZYX-nal11* gene, a G→T mutation occurs at the last base of the second exon, leading to an alteration in the alternative splicing pattern of the mRNA and abnormal translation [11]. In the *lw7* gene, a C→T mutation takes place at the 136th base of the coding sequence, forming a stop codon and causing premature translation termination [23]. For the *NAL11^−923del−1552^* gene, a fragment deletion occurs in the 923–1522 region of the promoter, resulting in an upregulation of the gene expression level [12].

HSP40 is also referred to as the DnaJ protein or J protein because it contains the DNAJ domain. Heat-shock proteins mainly function in abnormal protein degradation, protein three-dimensional structure folding, protein input and output, signal transduction, and transcriptional activation, and play important roles in the growth and development of plants and their stress resistance. According to the increasing molecular weight of the protein, they are mainly divided into the following six categories: sHSPs (15–42 kD), HSP40 (41 kD), HSP60 (60 kD), HSP70 (66–78 kD), HSP90 (83–90 kD), and HSP100.

When plants are exposed to adverse stress conditions such as high temperature, low temperature, drought, salt stress, and pathogen invasion, HSP40 is highly expressed and participates in stress resistance physiological and biochemical reaction processes. During the normal growth process of plants, HSP40 also participates in processes such as chloroplast development, photosynthesis, and protein transport [24]. There are a large number of genes encoding HSP40 in the rice genome [25], and the known cloned OsHSP40 genes in rice mainly play roles in salt stress resistance [26,27], biotic stress resistance [28,29,30], and chloroplast development [31], such as *LOC_Os02g01030* [26], *OsDNAJ15* [27], *OsDjA2* [28], *OsDjA6* [29], *OsDjA9* [30], and *LOC_Os05g26926* gene [31], While numerous leaf-shape regulatory genes have been identified, the research on the role of OsHSP40 gene in rice morphological development is relatively scarce. The complexity of leaf development—integrating internal structure, developmental processes, and environmental responses—necessitates further research to enrich our understanding of the regulatory network underlying rice leaf morphology [32].

Optimal plant architecture constitutes a fundamental prerequisite for breeding elite super rice cultivars, as this trait enables enhanced yield and grain quality. Superior genetic resources are essential for optimizing plant architecture [33]. The International Rice Research Institute (IRRI) established the Ideal Plant Architecture (IPA) concept, defined by the key traits of minimized unproductive tillers, robust culms, and enlarged panicles. During the past decade, Ideal Plant Architecture 1 (*IPA1*) has emerged as a key genetic target for enhancing rice yield. Fine-tuning *IPA1* expression optimizes plant architecture, inducing phenotypes of decreased tiller number, increased stem thickness, and larger panicle size, thereby demonstrating considerable utility in breeding programs [34]. Beyond its established function in the strigolactone (SL) pathway, IPA1 has been demonstrated to participate in gibberellin (GA) homeostasis regulation [35]. Impairment of *LOC_Os07g09450* gene function results in diminished chloroplast count, altered chloroplast morphology, and reduced thylakoid membrane abundance. These phenotypes, along with decreased tillering, are rescued by exogenous GA, which also improves emergence rate and seedling height in early growth stages [12]. Despite these, the mechanistic basis of IPA1-mediated plant architecture control through GA signaling cascades and associated downstream genes remains elusive. The present study provides a novel genetic resource for dissecting leaf-shape determination in rice, highlighting the role of the heat-shock protein OsHSP40 in regulating rice leaf morphology. These findings provide new insights and valuable resources for future research and targeted breeding aimed at optimizing rice plant architecture and increasing yield.

## Figures and Tables

**Table 1:** qPCR reaction system and reaction parameters.

Reagent	Usage/μL	Reaction Parameters				
2× Master Mix	10.0	Stage 1	Initial denaturation	Reps:1	95 °C	30 s
Primer1 (10 μM)	0.4	Stage 2	Cycle reaction	Reps:40	95 °C	3~10 s
Primer2 (10 μM)	0.4				60 °C	10~30 s
cDNA	0.2	Stage 3	Melting curve	Rep:1	95 °C	15 s
Sterilized double-distilled water	Add to 20.0				60 °C	60 s
					95 °C	15 s

**Table 2:** Primer sequences for fine-mapping.

Name	Forward Primer Sequence (5′-3′)	Reverse Primer Sequence (5′-3′)
RM7479	GCTCTGGTTAGTGATCATGG	ACATGGTGGCTTAGGAGTG
RM6574	AACCTCGAATTCCTTGGGAG	TTCGACTCCAAGGAGTGCTC
7-4915	ATTCAATCCAACCTCTCACAAT	CTTGAAGTTCTTGGGGTTCAT
7-4934	TACTGAGAGAGAGGGCTTGAGA	AACTAGTGGCATATTCGCTGAT
7-5037	TATGTGTGTGTGTGTGTGTGTG	TCAACAAATTTGGAAACAAAAA
7-5194	ATTAGCAGCAGCTTTGTATTCG	ATAGGTCCATGGTTGAACAAAA
7-5209	AGCATCGATAAGTTGAGGAGAC	GCCTGTTAGGAATGGGAAGT
7-5262	GGCTAAAAGTGGATAGATGCAG	ACACACCAAGATTGTCAGATCA
RM125	ATCAGCAGCCATGGCAGCGACC	AGGGGATCATGTGCCGAAGGCC
RM5672	CACCCTACAAGGAAACAAGC	TGCCCAATATAGAGGCAACC
R7M7	ACCTTCCCTCCCCTTTTGAT	AACTTGGTCTTCCTGTTTTATTG
RM6776	AGCCCGGACATGCAAAAC	GAAGCAGGCGAAATCTCCTC
RM1253	CTGAACTTGCCTGAGAACTC	GACGACCTCTCCATGCTCG
RM3718	AGCGCTCGAGAATTTCTAGG	ATGCTGACGTCACCCCAC
R7M20	GTTTTGTGCATTCCTTTAC	TTTATGACATTTGACCG
*Actin*-qPCR	CCAAGGCCAATCGTGAGAAGA	AATCAGTGAGATCACGCCCAG
*Nal25*-qPCR	ATTCTATGAAGGTGGCTTTCAA	TCCTTTTGTCTTCCCTAACAAA

**Table 3:** The flag leaf length/width and agronomic traits of the wild-type ‘Huazhan’ and the mutant *nal25*.

Agronomic Traits	Material	
	‘Huazhan’	*nal25*
Plant height/cm	108.00 ± 1.53	90.80 ± 2.48 ***
Flag leaf length/cm	36.57 ± 1.05	41.80 ± 1.66 **
Flag leaf width/cm	1.73 ± 0.05	0.57 ± 0.02 **
No. of effective panicles	25 ± 0.82	52 ± 1.63 ****
Panicle length/cm	25.63 ± 1.04	22.06 ± 1.33 ****
Total grain number of per spike	270.20 ± 5.53	75.25 ± 3.49 ****
Seed setting rate/%	94.25 ± 0.83	66.00 ± 2.92 ***
Grain length/cm	9.32 ± 0.18	8.97 ± 0.09 *
Grain width/cm	2.33 ± 0.05	2.09 ± 0.04 ***
1000-grain weight/g	19.78 ± 0.21	15.33 ± 1.52 *

Note: * significantly different at *p* < 0.05; ** significantly different at *p* < 0.01; *** significantly different at *p* < 0.001; **** significantly different at *p* < 0.0001.

**Table 4:** Genetic analysis on narrow and short leaf of *nal25* mutant.

Cross Combination	F_1_	F_2_			χ^2^ (χ^2^0.05 = 3.84)
		Total No. of Plants	No. of Wild-Type Plants	No. of Mutant-Type Plants	
*nal25*/Shenhui 26	normal	1145	862	283	0.05
*nal25*/Huazhan	normal	1192	905	287	0.46

## Data Availability

Data are contained within the article.

## Supplementary Materials

The following supporting information can be downloaded at https://www.mdpi.com/article/10.3390/plants14162528/s1: Table S1: 48 SSR markers for initial mapping of the *NAL25* gene.
