# Supplementary material for: Phenotypic Identification and Fine-Mapping of the Rice Narrow-Leaf Mutant nal25
Source: Plants (Basel). 2025 Aug 14;14(16):2528. doi: 10.3390/plants14162528 (PMC12389506; doi:10.3390/plants14162528)
Supplement: Supplementary file 1 [file plants-14-02528-s001.zip › plants-3762094-supplementary.pdf]

Supplementary information for 'Phenotypic Identification and Fine Mapping of the Rice Narrow Leaf Mutant nal25'

Supplementary Table S1. 48 SSR markers for initial mapping of the *NAL25* gene.

Number	Name	Forward primer sequence (5'-3')	Reverse primer sequence (5'-3')
1	RM583	AGATCCATCCCTGTGGAGAG	GCGAACTCGCGTTGTAATC
2	RM1195	ATGGACCACAAACGACCTTC	CGACTCCCTTGTTCTTCTGG
3	R1M30	AAGGGGCCCTAATTTATCTAG	TGTTTACTTTGTTCTTGGACTG
4	R1M37	ATAGTTCGCCATCGTCAT	ACACGCCATAGCAAGGAA
5	RM71	CTAGAGGCGAAAACGAGATG	GGGTGGGCGAGGTAATAATG
6	RM208	TCTGCAAGCCTTGTCTGATG	TAAGTCGATCATTGTGTGGACC
7	RM561	GAGCTGTTTTGGACTACGGC	GAGTAGCTTTCTCCCACCCC
8	R2M37	ACTGTTACCCAAACGCTA	ACGTGCACCTACTACAGAAA
9	RM85	CCAAAGATGAAACCTGGATTG	GCACAAGGTGAGCAGTCC
10	RM232	CCGGTATCCTTCGATATTGC	CCGACTTTTCCTCCTGACG
11	RM8277	AGCACAAGTAGGTGCATTTC	ATTTGCCTGTGATGTAATAGC
12	RM571	GGAGGTGAAAGCGAATCATG	CCTGCTGCTCTTTCATCAGC
13	R4M13	TACACGGTAGACATCCAACA	ATGATTTAACCGTAGATTGG
14	R4M17	AGTGCTCGGTTTTGTTTTC	GTCAGATATAATTGATGGATGTA
15	R4M30	GCTTCTCCTGGTTGTATGC	AAAATAGGGAGGCAGATAGAC
16	R4M50	TTTTGTGAAACTTGACCCTC	GCGTCCATGTCTTTATTGTG
17	RM274	CCTCGCTTATGAGAGCTTCG	CTTCTCCATCACTCCCATGG
18	RM267	TGCAGACATAGAGAAGGAAGTG	AGCAACAGCACAACTTGATG
19	R5M13	GAGAAAGAGTGGAAGGAG	AGTATCGTCAGGAGGGTC
20	R5M30	CTCAATTTCACCCATCCC	CGCTCCGTCTCCAACCTC
21	RM190	CTTTGTCTATCTCAAGACAC	TTGCAGATGTTCTTCCTGATG
22	RM253	TCCTTCAAGAGTGCAAAACC	GCATTGTCATGTCGAAGCC
23	RM176	CGGCTCCCGCTACGACGTCTCC	AGCGATGCGCTGGAAGAGGTGC
24	R6M44	TTAGGAATAAAGGCTGGATA	TTACCGTTAATAGGTGGAA
25	RM336	CTTACAGAGAAACGGCATCG	GCTGGTTTGTTTCAGGTTCG
26	RM481	TAGCTAGCCGATTGAATGGC	CTCCACCTCCTATGTTGTTG
27	RM542	TGAATCAAGCCCCTCACTAC	CTGCAACGAGTAAGGCAGAG
28	RM7479	GCTCTGGTTAGTGATCATGG	ACATGGTGGCTTAGGAGTG
29	R7M20	GTTTTGTGCATTCCTTTAC	TTTATGACATTTGACCG
30	RM542	TGAATCAAGCCCCTCACTAC	CTGCAACGAGTAAGGCAGAG
31	RM72	CCGGCGATAAAACAATGAG	GCATCGGTCCTAACTAAGGG
32	RM339	GTAATCGATGCTGTGGGAAG	GAGTCATGTGATAGCCGATATG
33	RM331	GAACCAGAGGACAAAAATGC	CATCATACATTTGCAGCCAG
34	RM219	CGTCGGATGATGTAAAGCCT	CATATCGGCATTCGCCTG
35	RM278	GTAGTGAGCCTAACAATAATC	TCAACTCAGCATCTCTGTCC
36	R9M30	CTCACCTACCTAAAACCCAAC	CCACCCAAATCTGATACTG
37	R9M42	CTATAAGACCAAAACGAAAACT	GAAAACCATTGTGTCACTGTA
38	RM311	TGGTAGTATAGGTACTAAACAT	TCCTATACACATACAAACATAC
39	RM258	TGCTGTATGTAGCTCGCACC	TGGCCTTTAAAGCTGTCGC
40	RM590	CATCTCCGCTCTCCATGC	GGAGTTGGGGTCTTGTTCG
41	RM209	ATATGAGTTGCTGTCGTGCG	CAACTTGCATCCTCCCCTCC
42	RM224	ATCGATCGATCTTCACGAGG	TGCTATAAAAGGCATTCGGG
43	RM21	ACAGTATTCCGTAGGCACGG	GCTCCATGAGGGTGGTAGAG
44	R11M23	AAGGTTGACAAGGACAGAAG	TCGCAGGAATGGATAAAA
45	RM19	CAAAAACAGAGCAGATGAC	CTCAAGATGGACGCCAAGA
46	RM17	TGCCCTGTTATTTTCTTCTCTC	GGTGATCCTTTCCCATTTCA
47	RM3331	CCTCCTCCATGAGCTAATGC	AGGAGGAGCGGATTTCTCTC
48	RM7102	TAGGAGTGTTTAGAGTGCCA	TCGGTTTGCTTATACATCAG
